# Identification of key interactions between SARS-CoV-2 main protease and inhibitor drug candidates

**DOI:** 10.1038/s41598-020-69337-9

**Published:** 2020-07-27

**Authors:** Ryunosuke Yoshino, Nobuaki Yasuo, Masakazu Sekijima

**Affiliations:** 1 Transborder Medical Research Center, University of Tsukuba, 1-1-1 Tennodai, Tsukuba, Ibaraki 305-8577 Japan; 2 Center for Computational Sciences, University of Tsukuba, 1-1-1 Tennodai, Tsukuba, Ibaraki 305-8577 Japan; 3 Tokyo Tech Academy for Convergence of Materials and Informatics (TAC-MI), Tokyo Institute of Technology, J3-23-4259 Nagatsutacho, Midori-ku, Yokohama, 226-8501 Japan; 4 School of Computing, Tokyo Institute of Technology, J3-23-4259 Nagatsutacho, Midori-ku, Yokohama, 226-8501 Japan

**Keywords:** Virtual drug screening, Drug screening, Infectious diseases, Computational biology and bioinformatics

## Abstract

The number of cases of severe acute respiratory syndrome coronavirus 2 (SARS-CoV-2) infection (COVID-19) has reached over 114,000. SARS-CoV-2 caused a pandemic in Wuhan, China, in December 2019 and is rapidly spreading globally. It has been reported that peptide-like anti-HIV-1 drugs are effective against SARS-CoV Main protease (M^pro^). Due to the close phylogenetic relationship between SARS-CoV and SARS-CoV-2, their main proteases share many structural and functional features. Thus, these drugs are also regarded as potential drug candidates targeting SARS-CoV-2 M^pro^. However, the mechanism of action of SARS-CoV-2 M^pro^ at the atomic-level is unknown. In the present study, we revealed key interactions between SARS-CoV-2 M^pro^ and three drug candidates by performing pharmacophore modeling and 1 μs molecular dynamics (MD) simulations. His41, Gly143, and Glu166 formed interactions with the functional groups that were common among peptide-like inhibitors in all MD simulations. These interactions are important targets for potential drugs against SARS-CoV-2 M^pro^.

## Introduction

In December 2019, numerous cases of pneumonia were reported in Wuhan, Hubei Province^1–3^ among which 19 confirmed cases and 39 imported cases were identified. The cause was identified as a new coronavirus disease (COVID-19) which is closely related to severe acute respiratory syndrome CoV (SARS-CoV)^4^. In early March, 88,913 cases of COVID-19 had been reported worldwide, 90% of the total were reported in China^5^, 8,739 cases of COVID-19 were reported to WHO from 61 countries outside of China, resulting in 127 deaths^5^. Moreover, The Republic of Korea has reported more than 4,200 cases and 22 deaths, which accounts for more than half of the cases of COVID-19 reported outside China^5^. To contain this virus outbreak, it is important to identify effective therapeutic drugs immediately^6^.

**Figure 1:**
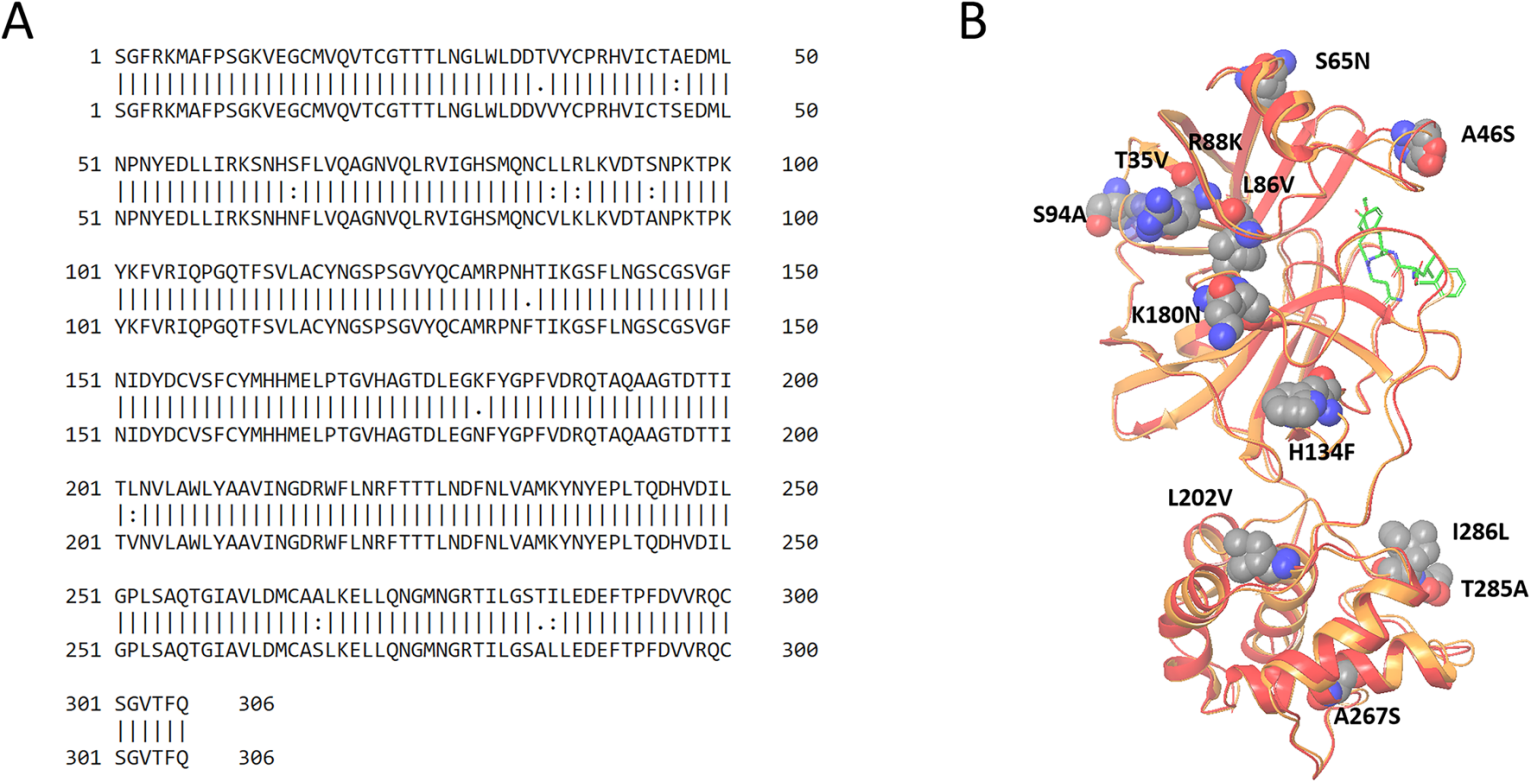
Alignment of SARS-CoV and SARS-CoV-2’s main protease sequences and X-ray structure. As a result of pairwise alignment, sequence identity showed 96%. The green stick model in (**B**) indicates the inhibitor binding site, and sphere model indicates residues that are not conserved between both sequences. (**A**) Pairwise alignment result of SARS-CoV M^pro^ (above sequence) and SARS-CoV-2 M^pro^ (below sequence), (**B**) Structure alignment result of SARS-CoV M^pro^ (PDB ID: 2A5I, red ribbon) and SARS-CoV-2 M^pro^ (PDB ID: 6LU7, orange ribbon).

SARS-CoV-2’s main protease (M^pro^), is emerging as a promising therapeutic target. This non-structural protein of coronavirus is responsible for processing the polyprotein translated from viral RNA^7^. It has been confirmed that viral replication is inhibited by M^pro^ inhibitor in SARS-CoV^8^. Its sequence is highly conserved with SARS-CoV M^pro^ (Fig. 1). When aligned, they show a sequence identity of 96%, and only the A46S mutation is located on the inhibitor binding site. Although no effective antivirals or vaccines against COVID-19 are currently reported, peptide-like HIV-1 protease inhibitors such as lopinavir and ritonavir have been reported to be effective against SARS-CoV M^pro^^8,9^. Clinical trials of these repurposed HIV protease inhibitors for COVID-19 have already been launched (e.g. ChiCTR2000029603, 2/6/20)^10^. However, the mechanism of action for SARS-CoV-2 M^pro^ at the atomic-level remains unknown. Understanding the mechanism of action at the atomic-level resolution may provide insights for more rational drug design^11^ and may decrease the risk of future drug resistance^12^.

Computational methods are commonly used for structure-based drug discovery (SBDD) and ligand-based drug discovery (LBDD)^13–18^. LBDD is a technique for searching and designing new drugs based on experimental information and structural information of known compounds^19,20^. On the other hand, SBDD is a method based on the tertiary structural information of the target protein^21^. This study focused on SBDD to discover three-dimensional insight for target binding. Pharmacophore modeling is one of LBDD techniques to discover common features of ligands to bind to the target protein^17^. Molecular dynamics (MD) simulations, in which the dynamics of biopolymers in solution can be analyzed at the atomic level, is a typical SBDD method used to predict the interaction between proteins and inhibitors^22–26^. MD simulation is based on Newton's equation of motion and has been applied to biomolecules such as proteins, nucleic acids, and lipid membranes^27–30^. Recent studies have shown that MD simulations can be applied to clarify the binding mechanism between proteins and compounds at the molecular level, which is highly useful for rational drug design^22–24,31–34^. Fortunately, many complex structures of SARS-CoV M^pro^ and inhibitor have already been determined and are available in the Protein Data Bank^35^. Therefore, by modeling the complex structure of SARS-CoV-2 M^pro^ and inhibitors using information on the known structure of SARS-CoV-M^pro^ and peptide-like inhibitors, it is possible to analyze the characteristics of functional groups required for the molecular recognition of ligands by SARS-CoV-2 M^pro^.

In the present study, we revealed important interactions for potential anti-coronavirus drugs to bind to SARS-CoV-2 M^pro^ by pharmacophore modeling and MD simulations. Based on pharmacophore modeling, three SARS-CoV-2 M^pro^ inhibitor candidates were selected, and SARS-CoV-2 M^pro^-inhibitor complex models were built. Subsequently, we conducted MD simulations for the SARS-CoV-2 M^pro^-inhibitor complex models to predict key characteristics of the functional groups required for molecular recognition by SARS-CoV-2 M^pro^ using interaction analysis.

## Methods

### Protein preparation and pharmacophore modeling

X-ray structures (2A5I, 2OP9, 6LU7) were downloaded from the Protein Data Bank (PDB). Assignment of bond orders and hydrogenation were performed using Maestro^36^. The suitable ionization states of each ligand were generated by Epik^37^ at pH 7.0 ± 2.0. Hydrogen bond optimization was performed using PROPKA^38^, and energy minimization calculations was conducted with Maestro using the OPLS3e force field^39^. Using the “protein structure alignment” tool in Maestro, all SARS-CoV M^pro^ structures were aligned to SARS-CoV-2 M^pro^ structure (PDB ID: 6LU7) to minimize RMSD based on alpha carbon. The pharmacophore was extracted by Phase^40,41^ using the conformation of the inhibitor in the structure of SARS-CoV M^pro^. After constructing the pharmacophore model, the protein of the SARS-CoV M^pro^-inhibitor complex superimposed on SARS-CoV M^pro^ was deleted, and the structure of the inhibitor and SARS-CoV-2 M^pro^ was merged. Indinavir was aligned to the pharmacophore model and the aligned Indinavir and SARS-CoV-2 M^pro^ structures were merged. Each merged structure was processed by hydrogen bond optimization and energy minimization calculations. These structures were used as initial structures for MD simulation.

### MD simulation

MD simulations for interaction analysis were performed using Desmond^42^. The inhibitor-SARS-CoV-2 M^pro^ complex models were placed in the orthorhombic box with a buffer distance of 10 Å in order to create a hydration model. TIP3P water model^43^ was used for creation of the hydration model. The cut-off radius for van der Waals and electrostatic interactions, time step, initial temperature and pressure of the system were set to 9 Å, 2.0 fs, 300 K and 1.01325 bar respectively. The sampling interval during the simulation was set to 50 ps. Finally, we performed MD simulations under the NPT ensemble for 1 μs using OPLS3e force field. Following MD simulations, the “Simulation Interactions Diagram” tool in Maestro was used to perform an interaction analysis between M^pro^ and inhibitor. Images of simulated proteins and ligands were generated using Maestro^36^.

## Results

### Structure alignment and pharmacophore modeling

**Figure 2:**
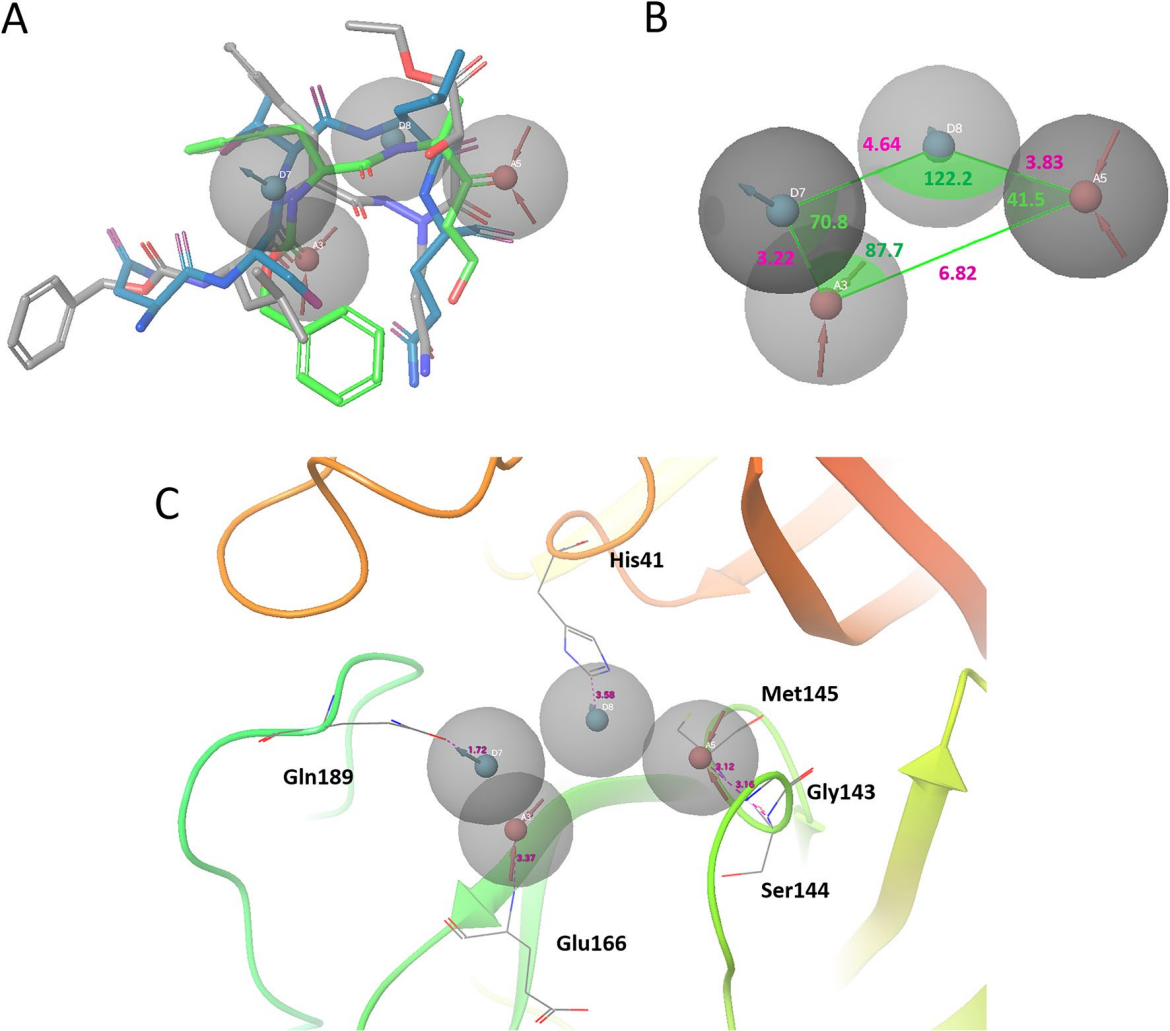
Pharmacophore model constructed by SARS-CoV M^pro^-inhibitor complex structure. Four features of inhibitors that bind to SARS-CoV M^pro^ were extracted. Blue spheres indicate H-bond donor (HBD), and red spheres indicates H-bond acceptor (HBA). (**A**) Alignment of pharmacophore model with each peptide-like inhibitor (Gray stick model: 2A5I ligand, Green stick model: 2OP9 ligand, Blue stick model: Indinavir). (**B**) Details of the positional relationship of the pharmacophore (Purple numbers: Distance between pharmacophores (Å), Green numbers: Angle between pharmacophores). (**C**) Amino acid residues of SARS-CoV M^pro^ (PDBID: 2A5I) around the pharmacophore model (His41-Donor sphere: 3.58 Å, Gly143-Acceptor sphere: 3.16 Å, Met145-Acceptor sphere: 3.12 Å, Glu166-Acceptor sphere: 3.37 Å, Gln189-Donor sphere: 1.72 Å).

To construct a SARS-CoV-2 M^pro^-inhibitor model, we performed structure alignment between SARS-CoV M^pro^-inhibitor complex structures and the SARS-CoV-2 M^pro^ structure. Figure 2A shows SARS-CoV M^pro^ inhibitors aligned with the pharmacophore model indicating the features of common functional groups of SARS-CoV M^pro^ inhibitors, namely 2A5I ligand and 2OP9 ligand, and Fig. 2B shows the positional relationship of the pharmacophore.

Using Phase software, two pharmacophore candidates, which were common among three ligands and had four pharmacophore points, were obtained (Fig. S1). These candidates had the same interactions, but slightly different 3D coordinates. It is because a pharmacophore is initially developed from single reference ligand by Phase algorithm, and two candidates were developed from different reference ligands. The pharmacophore that fits other active ligands more were chosen, by using (1) the root-mean-squared deviation (RMSD) in the pharmacophore point positions, and (2) the cosine of the angles formed by corresponding pairs of donor/acceptor. The total “screen score” (higher is better) of three active ligands are 5.34 and 5.07, respectively. The structural alignment and the pharmacophore model revealed that these inhibitors have two H-bond donor (HBD) functional groups and two H-bond acceptor (HBA) functional groups as common features. These features are located on the carbonyl oxygen atom and the amine, which forms peptide bonds in the backbone of peptide-like inhibitor. The blue stick molecule in Fig. 2A indicates the predicted conformation of indinavir to fit the pharmacophore model. Indinavir fits all four pharmacophore features built from the SARS-CoV M^pro^ inhibitor.

Figure 2C shows the amino acid residues around the chemical group defined as the pharmacophore. His41 and Gln189 are adjacent to the HBD sphere, and Gly143, Ser144, Cys145 and Glu166 are adjacent to the HBA sphere. His41’s side chain is located where the lone pair of nitrogen atoms on the imidazole ring can contact the donor sphere. Also, the carbonyl oxygen in the side chain of Gln189 is located near the donor sphere. These residues may form hydrogen bonds with the HBD located on the donor sphere. On the other hand, the HBA sphere is located near the main chain of Gly143, Ser144, and Cys145. The HBA sphere has a high affinity for the backbone NH Group. The backbone of Glu166 is also located near the HBA sphere, which enables NH group on the Glu166 backbone to connect with the HBA sphere. In Fig. 2C, these distance between His41, Gly143, Met145, Glu166, Gln189, and each pharmacophore sphere are 3.58 Å, 3.16 Å, 3.12 Å, 3.37 Å, 1.72 Å respectively.

### Interaction analysis by MD simulation

**Figure 3:**
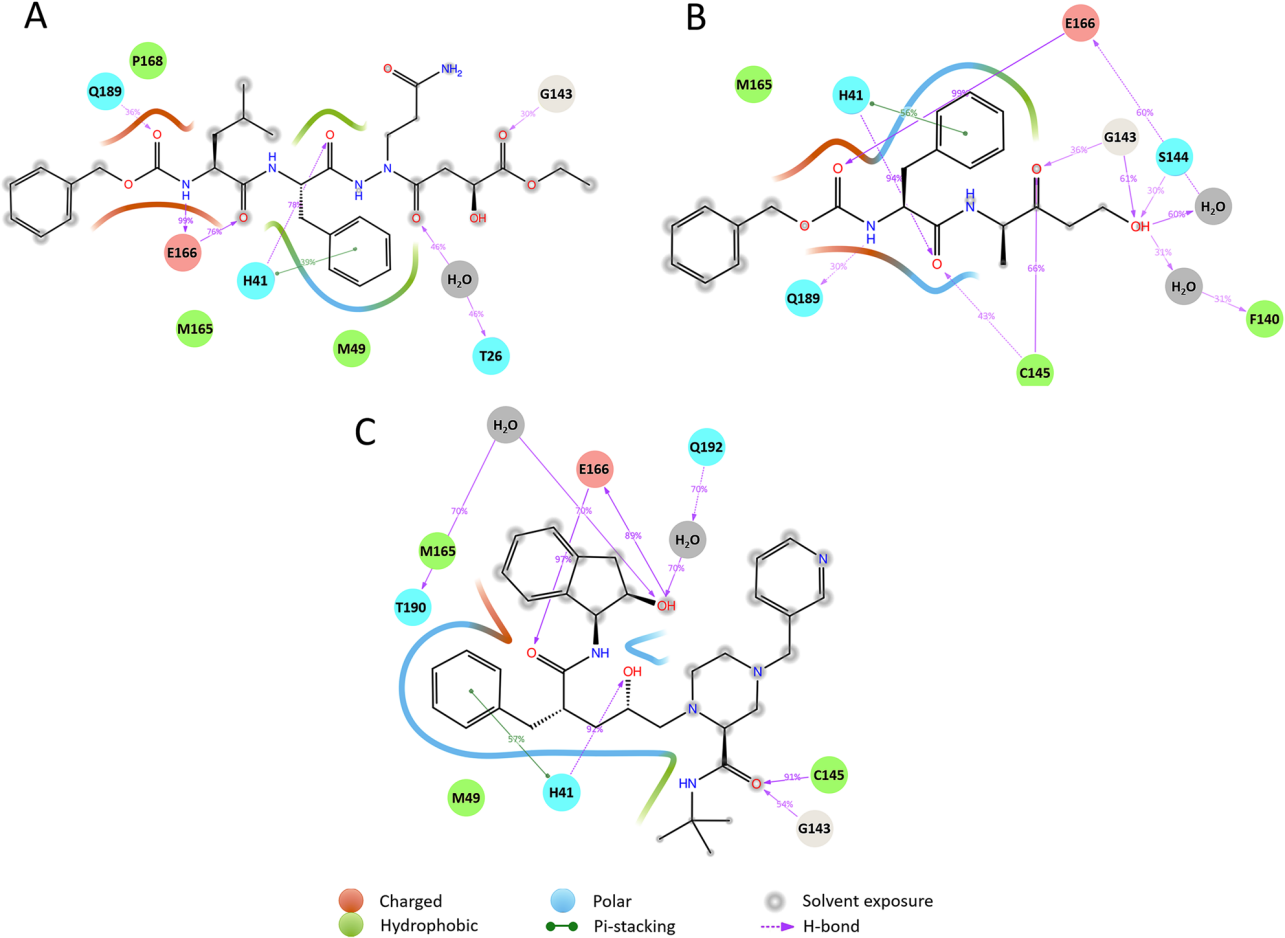
2D summary of the interaction analysis by MD simulation for each ligand. This figure contains SARS-CoV-2 M^pro^ amino acid residues which show an interaction probability of over 30% during MD simulation. Dotted lines indicate interactions between side chains and inhibitors, and solid lines indicate interactions between side chains and inhibitors. (**A**) Interaction results of 2A5I ligand, (**B**) Interaction results of 2OP9 ligand, (**C**) Interaction results of indinavir.

To clarify the key interactions between SARS-CoV-2 M^pro^ and drug candidates, we performed 1 μs MD simulations for each of six SARS-CoV-2 M^pro^-inhibitor complex models. The complex models were created by superimposing SARS-CoV M^pro^ into SARS-CoV-2 M^pro^. Protein and ligand RMSD information are presented in Figures S2 and S3. And root-mean-square fluctuation (RMSF) of amino acid residue is presented in Figure S4. Except for amino acid residues at both ends, the maximum RMSF of complex models is 2.0–2.4 Å (Figure S4A–C). In contrast, the maximum RMSF of apo form is 3.2 Å (Figure S4D). In the apo form result, fluctuations of amino acid residues around the 50th, 150th, and 270th positions are large (Figure S4D), and RMSF value around these regions decreases due to binding of inhibitor (Figure S4A–C). Figure 3 shows a 2D summary of the interaction analysis results of three SARS-CoV-2 M^pro^-inhibitor complex models. Timeline representation of the interactions and contacts are presented in Figure S5.

In all MD simulations, the interaction with Glu166 had the highest interaction rate. This residue mostly interacts with all ligands during each simulation (Figure S5). The 2A5I ligand and indinavir showed that it formed two hydrogen bonds with Glu166. Also, the interaction with His41 was maintained with a high probability in all MD results (78%, 92%, and 94%). This residue continues to interact with inhibitors during each simulation (Figure S5). Interactions with His41 were classified into two types: hydrogen bonding and Pi-stacking. In the interaction with His41, most of the hydrogen bond interactions were strongly connected. With the 2OP9 ligand and indinavir, hydrogen bonds to Gly143 and Cys145 were observed with a probability of over 50% during simulation. These interactions form with the main chain of Gly143 and Cys145. Two interactions were observed between the 2OP9 ligand and Cys145, in which the amine group of Cys145 main chain and the thiol group of Cys145 side chain were involved. With the 2A5I ligand and the 2OP9 ligand, an interaction between Gln189 and the inhibitors was confirmed with a probability of over 30% during simulation. 2A5I ligand, 20P9 ligand, and indinavir have one or two water bridge interactions with a probability of over 30% each during simulation. Especially, water between 2OP9 ligand and E166 forms a water bridge with a probability of 60%. According to the results of indinavir, water bridges are formed with T190 and Q192 with a probability of over 70%. Table 1 shows amino acid residues having an interaction probability of over 30% in each simulation. Interaction of His41, Gly143, Met165, and Glu166 were observed in all MD simulations. The side chains of His41 and the main chains of Gly143 and Glu166 were involved in the interaction, and Met165 forms a van der Waals (vdW) interaction with the inhibitors.

**Table 1:** Amino acid residues with interaction probability of over 30%.

2A5I ligand	2OP9 ligand	Indinavir
His41	His41	His41
Met49	Gly143	Met49
Gly143	Ser144	Gly143
Met165	Cys145	Cys145
Glu166	Met165	Met165
Pro168	Glu166	Glu166
Gln189	Gln189	

Table 2 shows the interactions probabilities related to pharmacophore during 1 μs MD simulation. Gly143-Acceptor and Met145-Acceptor are involved in the same pharmacophore point. Among pharmacophore interaction, His41-Donor and Glu166-Acceptor are highly stable during MD simulation for all compounds. Other interactions are also relatively stable except Met145-Acceptor of 2A5I ligand and Gln189-Donor of indinavir.

**Table 2:** Interaction probabilities related to pharmacophore during 1 μs MD simulation.

	2A5I ligand	2OP9 ligand	Indinavir
His41-donor	78%	94%	92%
Gly143-acceptor	30%	36%	54%
Met145-acceptor	–	66%	91%
Glu166-acceptor	99%	99%	97%
Gln189-donor	36%	30%	–

Gly143-Acceptor and Met145-Acceptor are involved in the same pharmacophore point.

–: less than 30% probabilities of interaction.

## Discussion

In this study, we first modeled a pharmacophore based on the structure of the SARS-CoV M^pro^ bound to peptide-like inhibitors. There were common features in the main chain of these peptide-like inhibitors. In Fig. 2C, SARS-CoV M^pro^ residues: His41, Gly143, Ser144, Cys145, Glu166, and Gln189 were located near these pharmacophore spheres. Since these residues are conserved in SARS-CoV-2 M^pro^, the features observed in SARS-CoV M^pro^ inhibitors will be located at similar positions in SARS-CoV-2 M^pro^ and thus, have the potential to inhibit SARS-CoV-2 M^pro^. Moreover, the three-dimensional structure of SARS-CoV M^pro^ and SARS-CoV-2 M^pro^ is almost conserved (Fig. 1B), and amino acid sequence identity value shows 96%. The pharmacophores do not contact unconserved amino acid residues in SARS-CoV M^pro^ and SARS-CoV-2 M^pro^. Thus, inhibitors that are matched with these pharmacophores may have the potential to inhibit both M^pro^.

**Figure 4:**
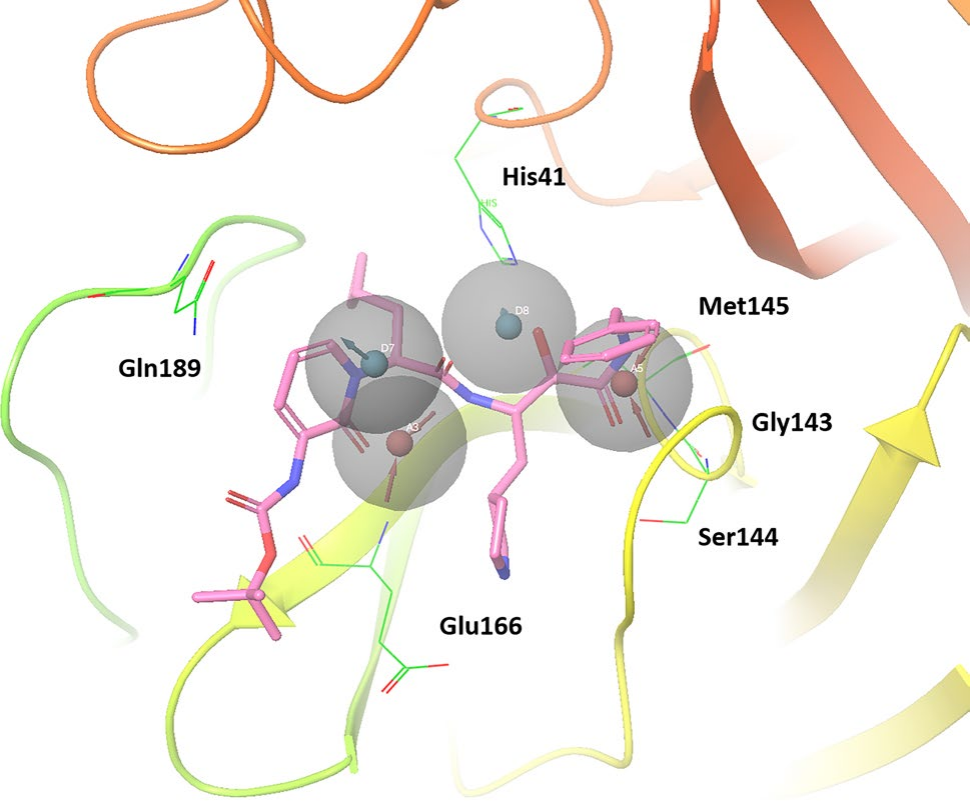
Alignment of α-ketoamide inhibitors and pharmacophore models. SARS-CoV-2 M^pro^ with α -ketoamide inhibitors (PDBID: 6Y2G) was aligned for 6LU7 and pharmacophore model using the “protein structure alignment” tool in Maestro.

To investigate the potential of these compounds to bind SARS-CoV-2 M^pro^, we performed MD simulations for SARS-CoV-2 M^pro^-inhibitor complex models. We observed strong hydrogen bonding with Glu166 main chain. In addition, although the thiol group of Cys145 interacts to the 2OP9 ligand, it was confirmed that the main chains of Gly143, Ser144, and Cys145 also interact with each inhibitor. It is suggested that the interaction with these amino acid residues may not be affected by side chain mutations unless the binding site shape or the dynamics of each chain are changed. Interactions with His41 were confirmed as hydrogen bonding and Pi-stacking. In the hydrogen bond, NH in the imidazole ring of His41 works as HBD. In addition, the imidazole ring of His41 also forms Pi-stacking with each inhibitor. According to the results of pharmacophore modeling, HBD pharmacophore sphere is located near His41. In contrast, the MD simulations suggested that His41 works as HBD. Therefore, HBA functional group has the potential to contact with His41. MD simulations also suggested that aromatic functional groups have high affinity for His41. In each MD simulation, Gly143, Ser144, Cys145, Glu166, and Gln189 interact with functional groups defined as pharmacophore of peptide-like inhibitors. Therefore, interactions with these amino acid residues are important for binding to SARS-CoV-2 M^pro^. In these MD simulation results, all ligand has one or two water bridges. Therefore, it is suggested that water bridges are involved in M^pro^ and inhibitor complex structure to stabilize the structure, functional groups of ligands can be extended to the space occupied by these waters. Figure 4 shows SARS-CoV-2 M^pro^ with α-ketoamide inhibitors (PDBID: 6Y2G)^44^ aligned to 6UL7. One hydroxyl group and two carbonyl groups of α-ketoamide are matched the pharmacophore model. However, one donor sphere is located at the nitrogen atom of the pyrimidine ring. Since this nitrogen atom has no hydrogen atom, it cannot function as a hydrogen bond donor. Comparing the structures of Gln189 in Figs. 2C and 4, the conformations of the side chains are different. Although the results of MD simulations suggested that the 2A5I ligand and the 2OP9 ligand interacted with Gln189, this structure has been suggested that the side chain conformation of Gln189 flexibly changes depending on the binding inhibitor. Irreversible inhibitors which have covalent bonds with Cys residue of SARS-CoV-2 M^pro^ have already been reported^44^. Irreversible inhibitors that selectively inhibit M^pro^ may have a higher binding affinity than competitive inhibitors and the inhibitors analyzed in this study are competitive inhibitors. However, drug repositioning is effective for highly urgent diseases such as COVID-19, and the pharmacophore proposed in this study can evaluate compounds which is not included a functional group to form a covalent bond with Cys. Therefore, the pharmacophore can be applicated for drug repositioning strategy.

In summary, this study suggests that compounds matching the pharmacophore model have potential as coronavirus inhibitors. Although these results were obtained from peptide-like inhibitors, the formation of these interactions allows the design and search of non-peptide-like compounds. The pharmacophore features that are important for binding to SARS-CoV-2 M^pro^ might help to develop new effective anti-coronavirus drugs.

## Supplementary information

Supplementary Figures.

## Data Availability

Initial X-ray structures are available at Protein Data Bank (https://www.rcsb.org/). Modeled structures for MD simulation are available at github (https://github.com/sekijima-lab/SARS-CoV-2_Mpro_structures). And the trajectory of all MD simulations can be downloaded from the following link (https://data.mendeley.com/datasets/5jfsx6j75g/2). The source data underlying Fig. 3A–C and Figs. S2A–C and S3A–C are provided as a Source Data file. Other data are available from the corresponding author upon reasonable request.
