# Supplementary material for: Identification of key interactions between SARS-CoV-2 main protease and inhibitor drug candidates
Source: Sci Rep. 2020 Jul 27;10:12493. doi: 10.1038/s41598-020-69337-9 (PMC7385649; doi:10.1038/s41598-020-69337-9)
Supplement: Supplementary file 1 — Supplementary Figures. [file 41598_2020_69337_MOESM1_ESM.pdf]

Supplementary Information for Identification of key interactions between SARS-CoV-2 Main Protease and inhibitor drug candidates

Ryunosuke Yoshino[1,2†], Nobuaki Yasuo[3†], Masakazu Sekijima[3*]

Affiliations

[1]Transborder Medical Research Center, University of Tsukuba, 1-1-1 Tennodai, Tsukuba, Ibaraki 305-8577, Japan

[2]Center for Computational Sciences, University of Tsukuba, 1-1-1 Tennodai, Tsukuba, Ibaraki 305-8577, Japan

[3]Advanced Computational Drug Discovery Unit, Tokyo Institute of Technology, J3-23-4259 Nagatsutacho, Midori-ku, Yokohama 226-8501, Japan

† These authors contributed equally to this study.

*Corresponding author

E-mail: sekijima@c.titech.ac.jp

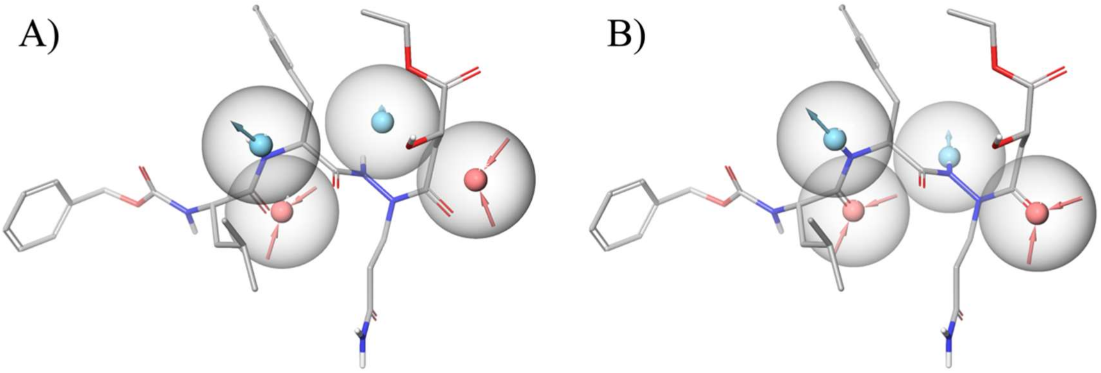

Figure S1. Pharmacophore model candidates constructed by SARS-CoV M^{pro}-inhibitor complex structure. Four features of inhibitors that bind to SARS-CoV M^{pro} were extracted. Blue spheres indicate H-bond donor (HBD), and red spheres indicates H-bond acceptor (HBA). Pharmacophore models were aligned with 2A5I ligand (Grey stick model). A: Used model (screen score: 5.34). B: Unused model (screen score: 5.07).

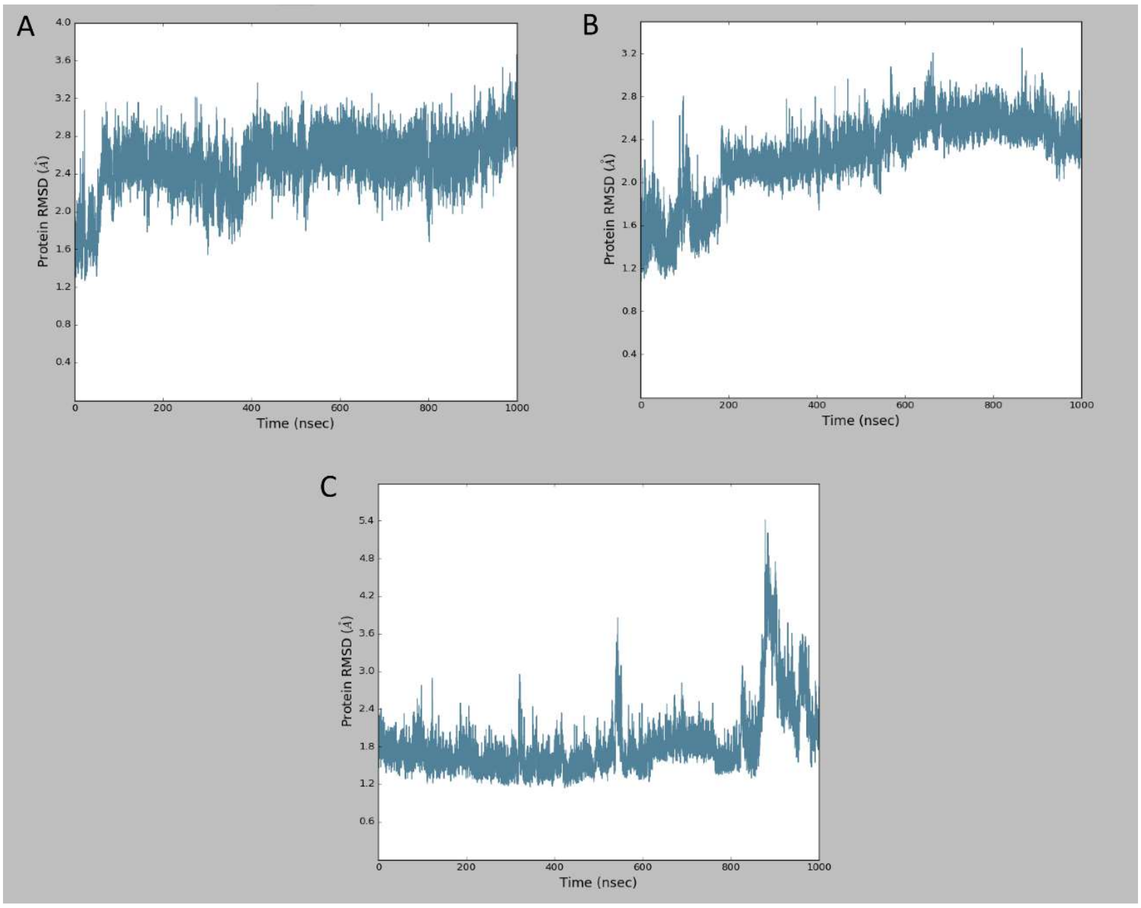

Figure S2. Root mean square deviations of SARS-nCoV-2 M^{pro} C_{α} atoms in the MD simulations. A: 2A5I ligand complex model, B: 2OP9 ligand complex model, C: Indinavir complex model

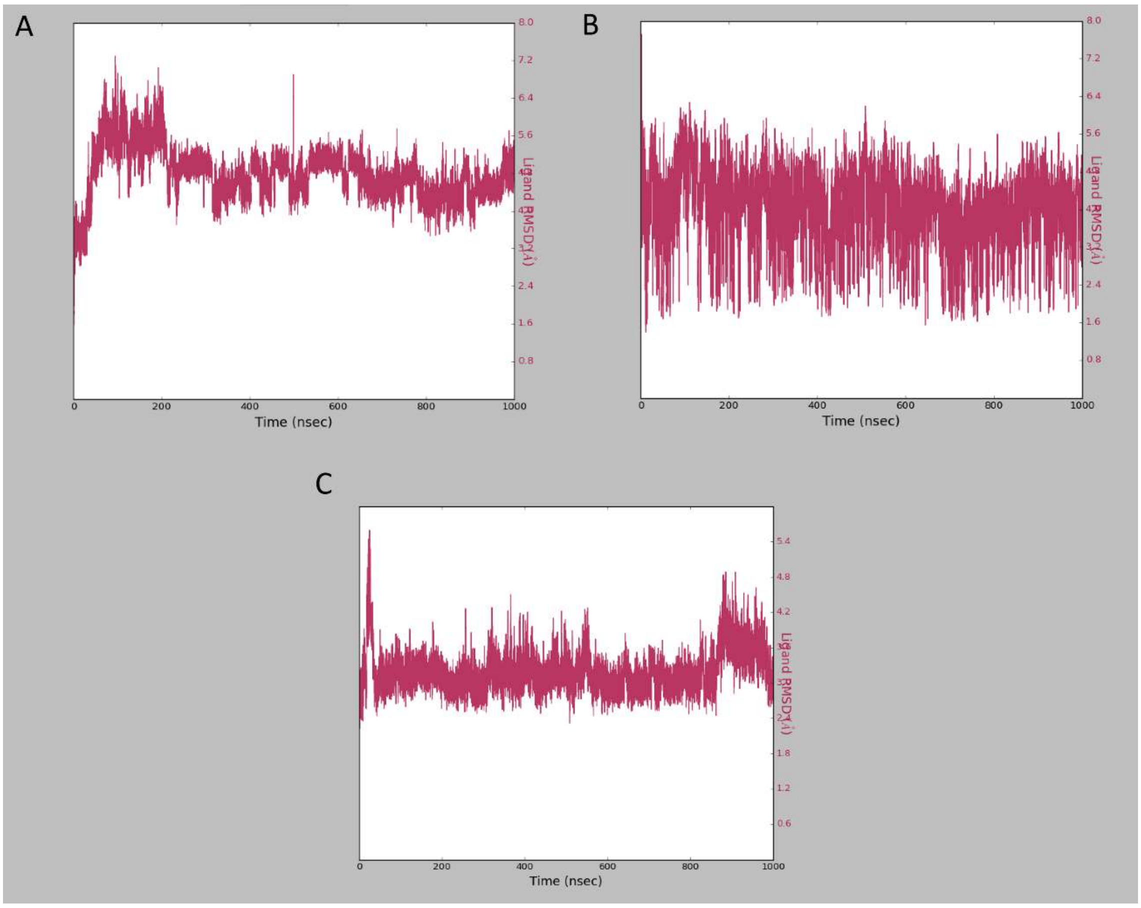

Figure S3. Root mean square deviations of ligand atoms in the MD simulations. A: 2A5I ligand, B: 2OP9 ligand, C: Indinavir

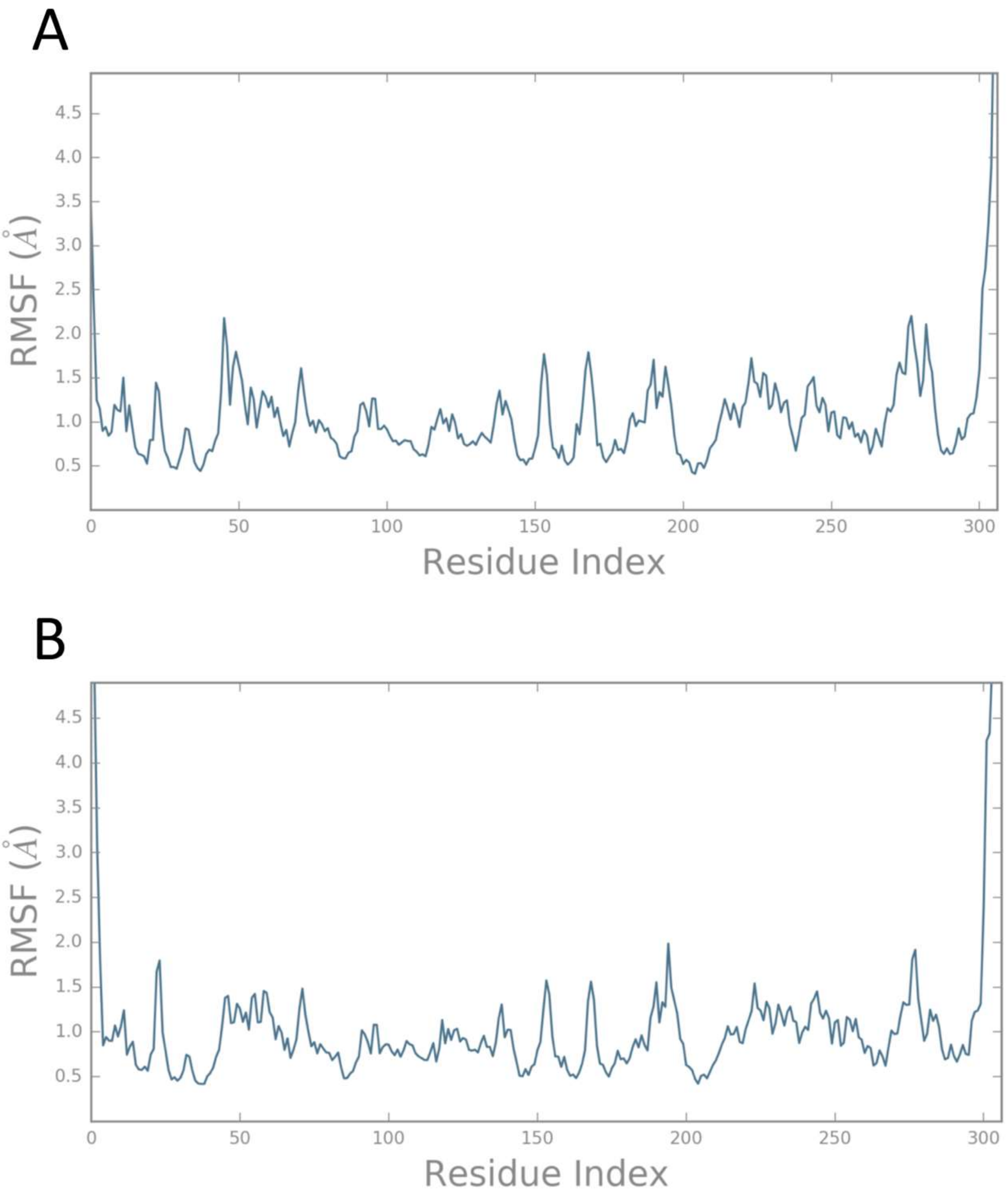

Figure S4. Root-mean-square fluctuation of amino acid residue in the MD simulations. A: 2A5I ligand, B: 2OP9 ligand

C

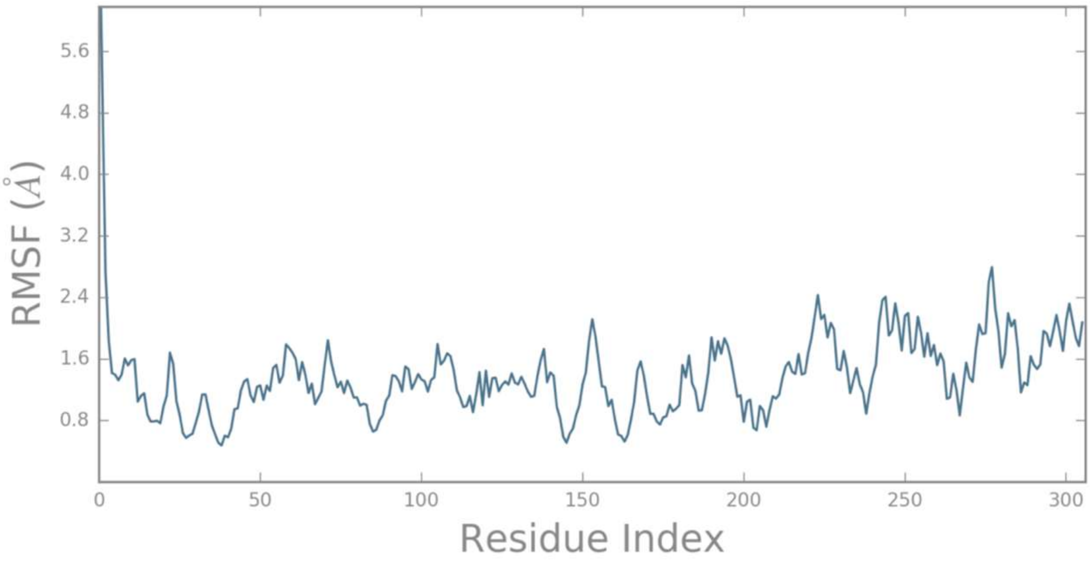

D

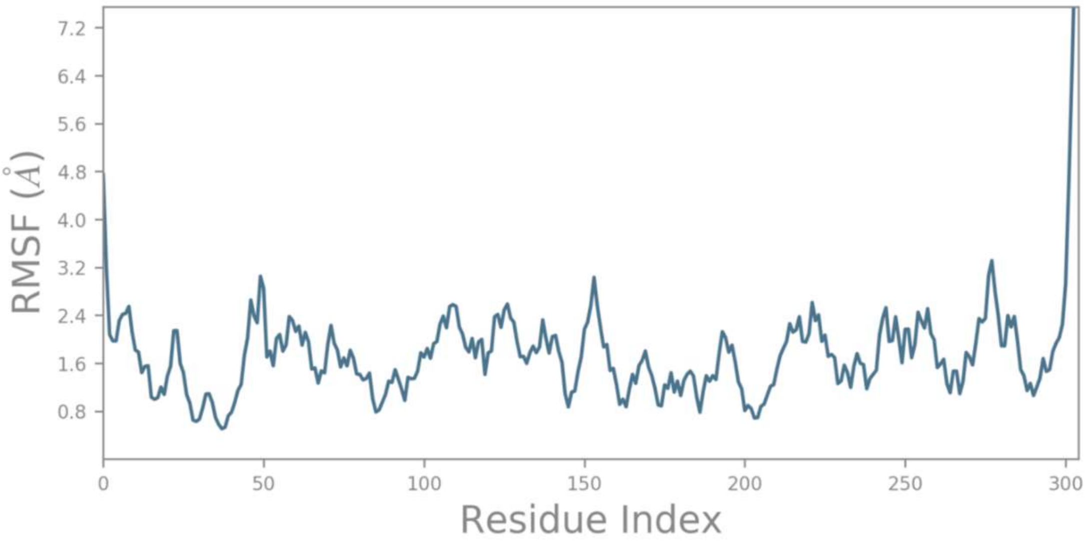

Figure S4. Continued. C: Indinavir, D: 6LU7 apo form

A

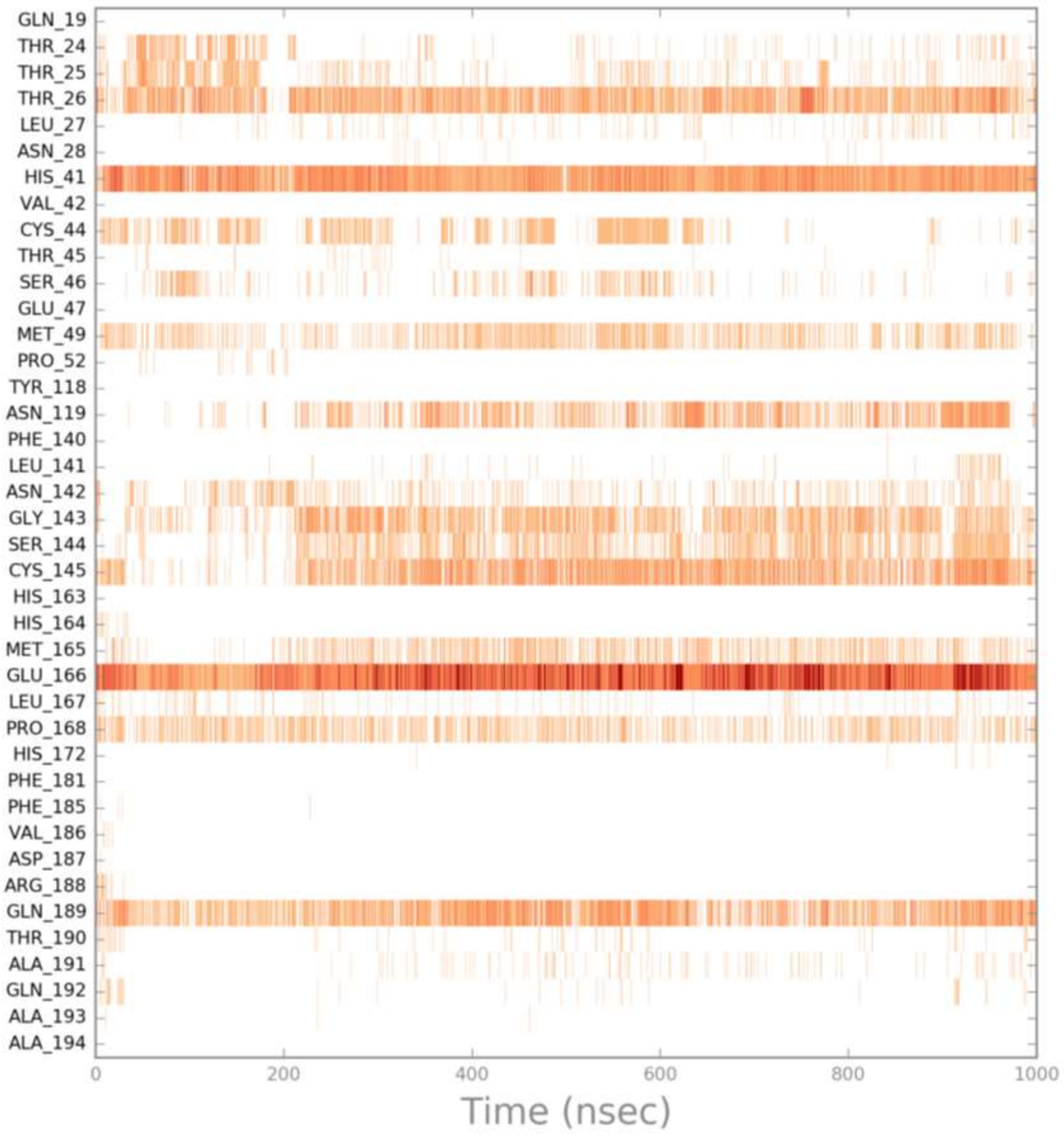

Figure S5. Timeline representation of the interactions and contacts (H-bonds, Hydrophobic, Ionic, Water bridges). This figure shows which residues interact with the ligand in each trajectory frame. Some residues make more than one specific contact with the ligand, which is represented by a darker shade of orange. A: 2AI5 Ligand.

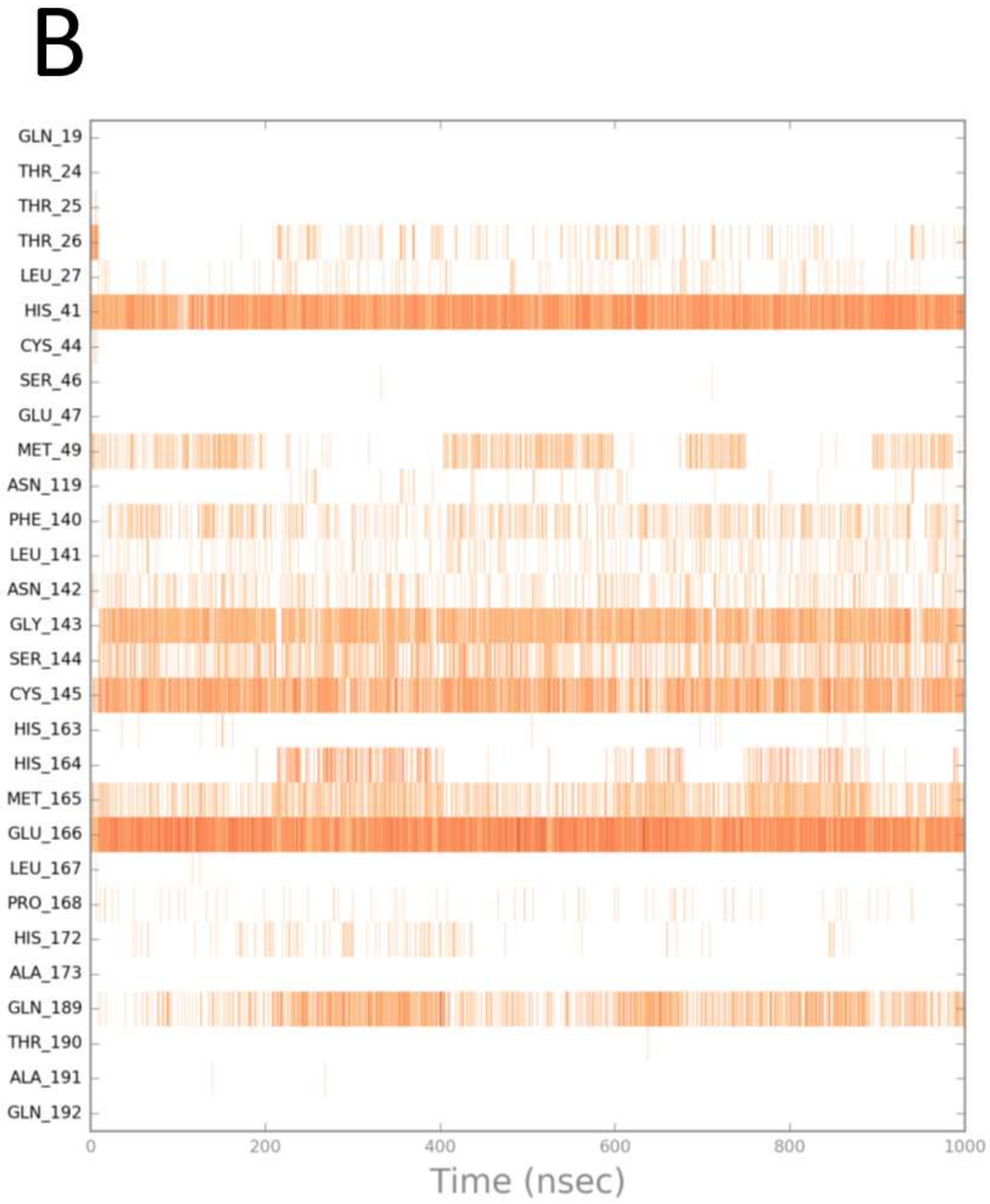

Figure S5. Continued. B: 2OP9 Ligand.

C

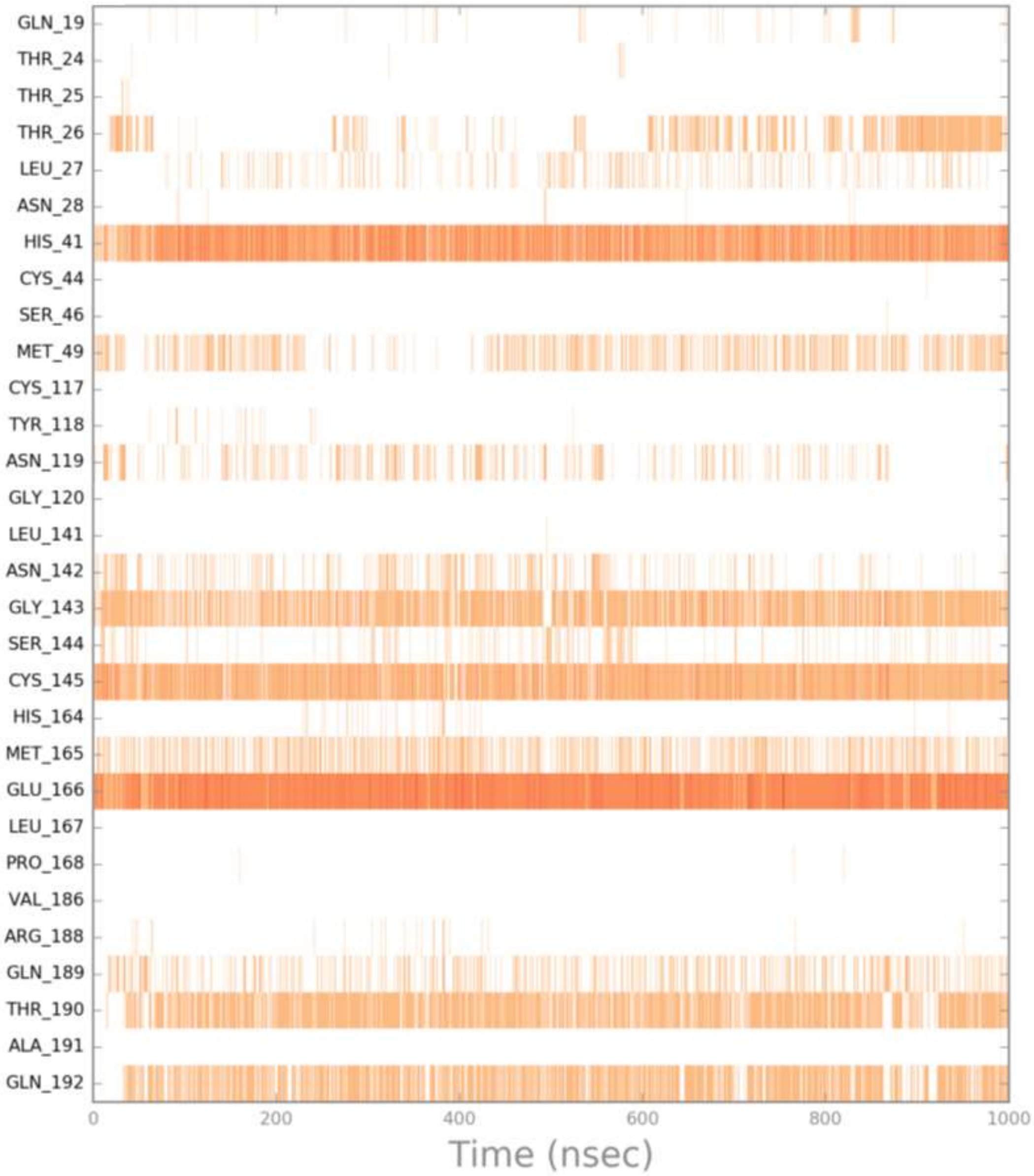

Figure S5. Continued. C: Indinavir.
